# Androgen receptor (AR) signaling promotes RCC progression *via* increased endothelial cell proliferation and recruitment by modulating AKT → NF-κB → CXCL5 signaling

**DOI:** 10.1038/srep37085

**Published:** 2016-11-16

**Authors:** Zhenfeng Guan, Chong Li, Jinhai Fan, Dalin He, Lei Li

**Affiliations:** 1 Department of Urology, The First Affiliated Hospital, Xi’an Jiaotong University, Xi’an 710061, China; 2 Core Facility for Protein Research, Institute of Biophysics, Chinese Academy of Sciences, Beijing 100101, China; 3 Beijing Jianlan Institute of Medicine, Beijing 100190, China

## Abstract

Androgen receptor (AR) signaling may promote renal cell carcinoma (RCC) progression *via* altered HIF-2α/VEGF signaling. However, it remains unclear whether AR signaling also promotes RCC progression by recruiting vascular endothelial cells (ECs), key players in the development of blood vessels. In our study, AR increased EC proliferation and recruitment to the tumor microenvironment and promoted RCC progression. Mechanistically, AR modulated cytokine CXCL5 expression by altering AKT → NF-κB signaling, and interruption of AKT → NF-κB → CXCL5 signaling using either specific inhibitors or siRNA suppressed AR-enhanced EC recruitment and AR-EC-promoted RCC progression. The results obtained using an *in vivo* mouse model and a human clinical sample survey confirmed the role of AR in promoting RCC progression through enhancement of EC proliferation and/or recruitment *via* altered AKT → NF-κB → CXCL5 signaling. Targeting this newly identified AR-induced AKT → NF-κB → CXCL5 pathway may facilitate the development of new therapies for slowing RCC progression.

The worldwide incidence of renal cell carcinoma (RCC) has steadily increased over the past two decades^1^,^2^. Approximately 20–30% of patients with RCC are diagnosed with metastatic lesions, and nearly 20% of post-surgery patients will relapse and develop metastatic RCC (mRCC)^2^,^3^. Microvessel formation is a key factor during RCC progression, especially in mRCC patients^4^,^5^. Although targeting tumor angiogenesis is a standard therapeutic strategy for mRCC, the mechanisms through which it occurs remain unclear.

Tumors induce and sustain the growth of new blood vessels through angiogenesis^6^, and blood vessels within a tumor are heterogeneous, highly permeable, chaotically branched, and often described as abnormal or dysfunctional^7^. Endothelial cells (ECs), which are the main components of blood vessels and play prominent roles in the initial phases of tumor angiogenesis, can be recruited from the bone marrow and circulation by angiogenic factors such as vascular endothelial growth factor (VEGF) and angiopoietin-1^8^. Chemokines, key inflammatory factors, promote EC recruitment and RCC progression^9^,^10^,^11^. According to numerous studies, nuclear factor-κB (NF-κB) signaling plays a central role in chemokine expression and is involved in tumorigenesis and cancer cells inflammation^12^, and NF-κB could be a key downstream component of PI3K/AKT signaling^11^. Clinically, targeting the PI3K/AKT pathway has yielded good outcomes for RCC patients. However, the detailed mechanisms of PI3K/AKT/NF-κB signaling during RCC EC recruitment are not yet understood.

Androgen receptor (AR) signaling is crucial during prostate cancer initiation and progression^13^. According to a recent study, AR is expressed in 30% of RCC tissues^14^, and He *et al.* also demonstrated that AR signaling promotes RCC progression *via* modulation of HIF-2α/VEGF signaling^15^. Nonetheless, the potential link between AR signaling and blood vessel formation/development or angiogenesis remains unclear.

Here, we demonstrate that AR signaling promotes RCC progression *via* increased endothelial cell proliferation and recruitment by modulating AKT → NF-κB → CXCL5 signaling.

## Materials and Methods

### Cell culture and stable cell lines

Human RCC cell lines 786-O, 769-P (AR-negative cells, AR^−^) and OS-RC-2 (AR-positive cells, AR^+^) were obtained from ATCC (American Type Culture Collection, *USA*). The 786-O and 769-P cells were maintained in RPMI-1640, whereas the OS-RC-2 cells were maintained in Dulbecco’s modified Eagle’s medium (DMEM); both media (Invitrogen, Carlsbad, CA, *USA*) were supplemented with 10% fetal bovine serum (FBS) (Invitrogen, Carlsbad, CA, *USA*). Cells were grown in a 5% CO_2_ and 37 °C incubator. Stable clones of OS-RC-2 cells with AR knock-down and AR-over-expressing 769-P and 786-O cells were transfected with siRNA or AR lentivirus as described in our previous study^16^.

### Chemical reagents, inhibitors and antibodies

DHT (dihydrotestosterone) and Casodex (Bicalutamide) (Sigma-Aldrich, *USA*) were applied in *vitro* to activate or inhibit AR signaling at final concentrations of 10 nM and 1 μM, respectively. siRNA was used to knock-down CXCL5, P65 and P110 in RCC samples. Anti-GAPDH (6c5), -β-actin (I-19), -AR (N-20), and - tetramethylrhodamine isothiocyanate (TRITC) IgG antibodies were purchased from Santa Cruz Biotechnology. Anti-CD31 and -P65 antibodies were obtained from Millipore. 5-Bromo-2-deoxyuridine (BrdU) and crystal violet were obtained from Fisher Scientific. Anti-mouse/rabbit secondary antibodies for western blotting were obtained from Invitrogen.

To functionally inhibit/activate potential signaling pathways, we utilized LY29400/IGF-1 (a specific inhibitor/activator, respectively, of the PI3K/Akt pathway) and PDTC/TNF-α (a specific inhibitor/activator, respectively, of NF-κB signaling). To determine the role of CXCL5 in EC recruitment, before analysis, we applied a CXCL5 neutralizing antibody for 1 hr at room temperature at a final dilution of 1:300.

### Cell migration, invasion and recruitment assays

Twenty-four-well (8 μm pores) transwell plates (Millipore, *Switzerland*) were used for migration and invasion assays. For *in vitro* invasion assays, the upper chambers of the transwells were pre-coated with diluted Matrigel (Dilution ratio: 1:4. Matrigel, BD Biosciences, Sparks, MD). CM (conditioned medium) was obtained by co-culturing HUVECs with RCC cells. Briefly, the two types of cells were cultured in the same dish for 24 hr, and the supernatants were collected and filtered to remove cells.

Before performing invasion assays, RCC cells were treated with CM for 48 hr. First, 10^4^ CM-treated RCC cells (serum-free) and serum-free medium were plated in the upper and lower chambers, respectively. After 36 hr of incubation, invaded cells were stained with 0.1% crystal violet and counted. The cell numbers were obtained by averaging the counts from 5 random fields. The migration assay was performed using the same approach as the invasion assay (omitting Matrigel) with an incubation time of 24 hr. The data are presented as triplicate repeats ± SEM.

The ability of RCC to recruit ECs was monitored using recruitment assays. Briefly, ECs (HUVECs) were plated in the upper chamber (with 8 μm pores), and RCC cells were plated in the lower chamber.

### Cell proliferation assay

BrdU incorporation was used to demonstrate RCC cell/EC proliferation. Briefly, RCC cells/ECs were seeded into 24-well plates and allowed to reach 50–70% confluence. BrdU was added to the medium for 4 hr (3 μg/ml), and the cells were fixed with 4% paraformaldehyde. Then, 0.1% Triton X-100 was used to destroy the cell membrane (15 min), and 2 N HCl (25 min) was used to separate the DNA into single strands. The cells were incubated in 10% bovine serum albumin (BSA) with an anti-BrdU antibody (1:200) overnight at 4 °C, followed by incubation with a TRITC-labeled secondary antibody for 1 hr at RT. The fluorescence intensity of TRITC was monitored using a Super Micro Orifice Plate Spectrophotometer (BioTek, USA) at 547 nm.

### RNA extraction and Q-PCR analysis

Total RNA was isolated using TRIzol reagent (Invitrogen, Carlsbad, CA, *USA*), and 2 μg of total RNA was subjected to reverse transcription using Revert Aid TM First-Strand cDNA Synthesis Kit (MBI Fermentas, St. Leon-Rot, *Germany*) according to the manufacturer’s protocol. Quantitative-PCR was conducted using a Bio-Rad CFX96 system with SYBR green to determine the mRNA expression level of genes of interest. Expression levels were normalized to the expression of GAPDH RNA.

### Western blot analysis

Expression of AR and related proteins was determined by western blotting according to a previous study^16^. In brief, total cellular protein lysates were prepared with RIPA buffer [50 mM Tris (pH 8.0), 150 mM NaCl, 0.1% SDS, 1% NP40 and 0.5% sodium deoxycholate] containing proteinase inhibitors [1% cocktail and 1 mM PMSF, both from Sigma, (St. Louis, MO, *USA*)]. A total of 30 μg of protein was separated by 8–10% SDS-PAGE and transferred to nitrocellulose membranes. After blocking, the membranes were incubated with the appropriate dilutions (1:1000) of specific primary antibodies. Next, the blots were incubated with HRP-conjugated secondary antibodies and visualized using Odyssey Detection System (Licor, Rockford, IL, *USA*).

### Immunofluorescence staining for nuclear translocation of NF-κB

Immunofluorescence staining was performed as described previously^17^. In brief, cells on slides were washed three times with cold phosphate-buffered saline (PBS) (pH 7.4) and then fixed with 4% paraformaldehyde for 15 min, permeabilized in 0.5% Triton X-100 for 10 min, and incubated in 1% BSA blocking solution for 1 hr. The fixed cells were incubated overnight at 4 °C with rabbit anti-human-P65 (1:250) in 1% BSA. The cells were washed and incubated with a mouse anti-rabbit TRITC (red) IgG antibody (Santa Cruz, *USA*) (diluted 1:100 in blocking buffer) for 1 h. Nuclei were stained with 4′,6-diamidino-2-phenylindole (DAPI) for 5 min. The cells were examined using a fluorescent microscope equipped with narrow band-pass excitation filters to individually select for red and blue fluorescence. The cells were observed using Image Pro Plus System^®^ mounted on a fluorescent microscope (Olympus, *Japan*). All experiments were performed in triplicate.

### Renal capsule implantation and *in vivo* vascular endothelial cell recruitment assay

To demonstrate the effect of vascular ECs on RCC tumorigenesis, RCC cells plus or minus HUVECs were implanted into mouse kidney capsules. In brief, 10^6^ RCC cells (including 769-P and OS-RC-2) were co-cultured with *human umbilical vein endothelial cells* (HUVECs) for several weeks. The co-cultured RCC cells were then injected into the kidney capsules of 8 nude mice per group. After 4 weeks, the tumor masses were harvested, weighed, fixed with 4% formalin, and prepared for *hematoxylin* and eosin (HE) staining. The mouse care and protocols were approved by the Institutional Animal Care and Use Committee of Xi’an Jiaotong University, and all mouse experiments were performed in adherence with the National Institutes of Health Guidelines on the Use of Laboratory Animals.

For HUVEC recruitment assays *in vivo*, 10^6^ RCC cells (including 769-P and OS-RC-2) were injected into the kidney capsules of nude mice for at least 4 weeks to assess tumorigenesis. HUVECs were tagged by luciferase via a tail vein injection 24 hr before tumor harvest. Further immunohistochemistry (IHC) staining was used to detect the luciferase-tagged HUVECs within the tumor masses.

### HE and IHC staining

For HE staining, tissue sections were de-waxed and rehydrated using routine methods. The sections were stained with hematoxylin for 5 min and washed in running tap water for 5 min. The sections were then stained with eosin for 30 sec, dehydrated, and mounted using routine methods. Figures show representative fields.

IHC staining was conducted using Image Pro Plus System (Olympus, *Japan*). A rabbit polyclonal antibody against AR (N-20, 1:500 diluted) and anti-CD31 (H-300, 1:300 diluted) and anti-luciferase antibodies were used. Slides were further analyzed. Five random fields from each slide were recorded.

### Statistical analyses

ANOVA was performed to compare three or more groups. Student’s *t*-test was performed to detect significant differences between two groups. P values <0.05 were considered significant.

## Results

### AR signaling increases EC proliferation and recruitment to RCC

Early studies^15^,^18^,^19^ suggested that AR signaling promotes RCC progression by modulating HIF-VEGF signaling. Interestingly, other studies have also indicated that ECs, the key components that contribute to the formation/development of blood vessels, might also play important roles in the progression of various tumors, including RCC^9^,^10^. However, the link between AR signaling and ECs in RCC progression has remained unclear.

**Figure 1:**
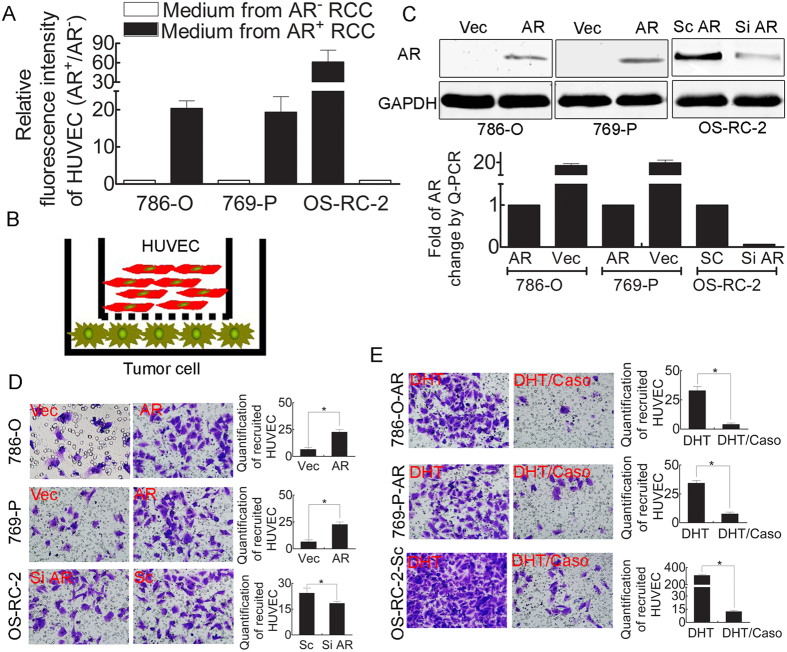
Activation of AR signaling in RCC plays a vital role in HUVEC proliferation and recruitment. (**A**) CM from AR^+^ RCC cells (786-O-AR, 769-P-AR and OS-RC-2-Sc AR) resulted in enhanced HUVEC proliferation compared to that from AR^−^ RCC cells (786-O-Vec, 769-P-Vec and OS-RC-2-Si AR) (as demonstrated by the BrdU incorporation assay). (**B**) Cartoon illustrating the HUVEC recruitment assay. (**C**) The efficiency of AR overexpression (786-O and 769-P) or knock-down (OS-RC-2) assayed by western blotting and Q-PCR. (**D**) Boyden Chamber assay: addition of AR to 786-O/769-P cells or AR knock-down in OS-RC-2 cells enhanced or attenuated HUVEC recruitment, respectively. Left: representative figures. Right: quantification of the Boyden Chamber results. Bar: 100 μm. *P < 0.05. (**E**) Boyden Chamber assay: addition of 10 nM DHT significantly enhanced HUVEC recruitment by AR^+^ RCC cells, including 786-O-AR, 769-P-AR and OS-RC-2-Sc AR cells. These effects were attenuated with 1 μM Casodex. Left: representative figures. Right: quantification of the Boyden Chamber data. Bar: 100 μm. *P < 0.05.

To study the potential roles of AR signaling in modulating ECs in RCC, we first utilized the BrdU incorporation assay to determine the impact of AR signaling on EC proliferation during the co-culture of RCC AR^+^ cells with HUVECs. CM from RCC AR^+^ cells significantly promoted HUVEC proliferation compared with CM from RCC AR^−^ cells (Fig. 1A).

Because an increased number of ECs may also be recruited from the RCC tumor microenvironment (TME)^20^, we also utilized a Boyden Chamber assay to examine the effect of AR signaling on the recruitment of ECs to RCC. The results from a recruitment assay with co-cultured HUVECs and RCC cells (Fig. 1B) with either AR added or AR knock-down (Fig. 1C) demonstrated that adding AR to RCC 769-P cells (769-P-AR) and 786-O cells (786-O-AR) increased recruitment of HUVECs to RCC cells. AR knock-down in RCC OS-RC-2 cells (OS-RC-2-Si AR) using siRNA decreased recruitment of HUVECs to RCC cells (Fig. 1D). Furthermore, addition of 10 nM DHT to 786-O-AR, 769-P-AR and OS-RC-2 cells resulted in greater recruitment of HUVECs to RCC, and these increases could be reversed/abolished by adding 1 μM of the anti-androgen Casodex (Fig. 1E).

Taken together, the results shown in Fig. 1A–E, using either proliferation or recruitment assays, indicate that AR signaling increases the number of ECs recruited to RCC.

### Mechanism: how does AR signaling increase recruitment of ECs to RCC?

The potential molecular mechanism(s) by which AR signaling enhances EC recruitment to RCC is unknown. To address this, we first utilized a Q-PCR-based super-array analysis to search for key genes that link AR function to EC recruitment. We found that adding AR to 786-O and 769-P cells or that AR knock-down in OS-RC-2 cells significantly increased or decreased expression of several cytokines (Fig. S1A). Among these inflammation-related cytokines, we focused on CXCL5 because it contributes to tumor metastasis and recurrence of intrahepatic cholangiocarcinoma by recruiting infiltrative intratumoral neutrophils^21^. We then utilized the Q-PCR assay to confirm that AR altered CXCL5 mRNA expression in both RCC 769-P-AR and OS-RC-2 cells (Fig. S1B).

**Figure 2:**
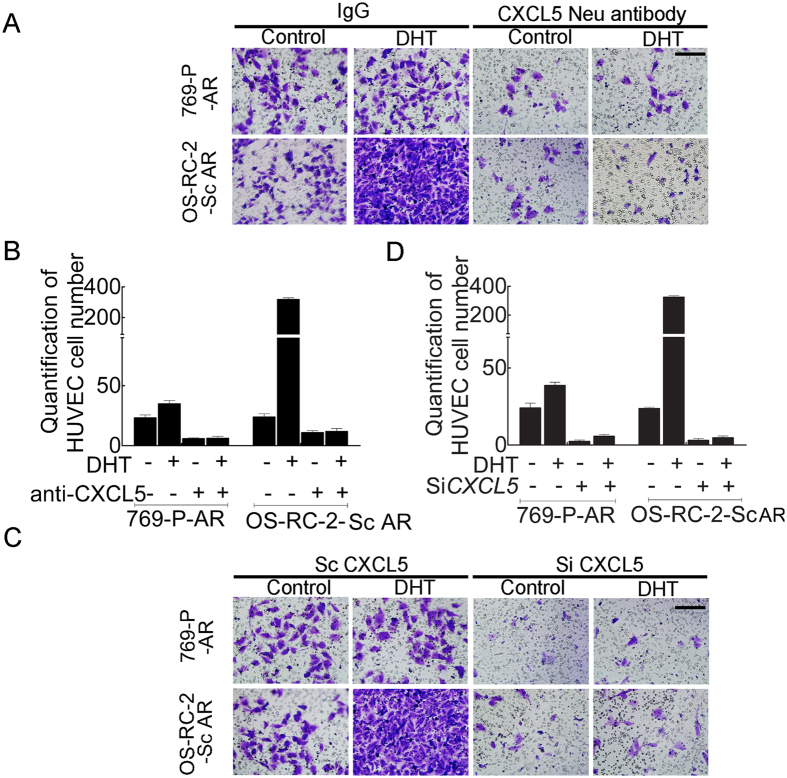
Inhibition of *CXCL5* expression in AR-positive RCC cells decreases HUVEC recruitment. (**A**) Functional inhibition of CXCL5 using a neutralizing antibody in 769-P-AR and OS-RC-2-Sc AR cells led to decreased HUVEC recruitment according to the Boyden Chamber assay. Bar: 100 μm. (**B**) Quantification of Fig. 2A. (**C**) CXCL5 knock-down in 769-P-AR and OS-RC-2-Sc AR cells using siRNA attenuated HUVEC recruitment according to the Boyden Chamber assay. Bar: 100 μm. (**D**) Quantification of Fig. 1C results.

Importantly, using interruption approaches with a specific CXCL5 neutralizing antibody, we observed that blocking CXCL5 reduced recruitment of HUVECs to RCC 769-P-AR and OS-RC-2 cells in the presence or absence of DHT (Fig. 2A,B). Similar results were also obtained when we replaced the anti-CXCL5 antibody with CXCL5 shRNA to suppress CXCL5 in RCC 769-P-AR and OS-RC-2 cells. Thus, CXCL5 knock-down abolished recruitment of HUVECs to RCC in the presence or absence of DHT (Fig. 2C,D).

Overall, the results presented in Fig. 2A–D demonstrate that AR signaling might function by altering CXCL5 expression to enhance EC recruitment to RCC cells.

### AR-modulated CXCL5 functions by activating AKT/NF-κB signaling to enhance EC recruitment to RCC cells

**Figure 3:**
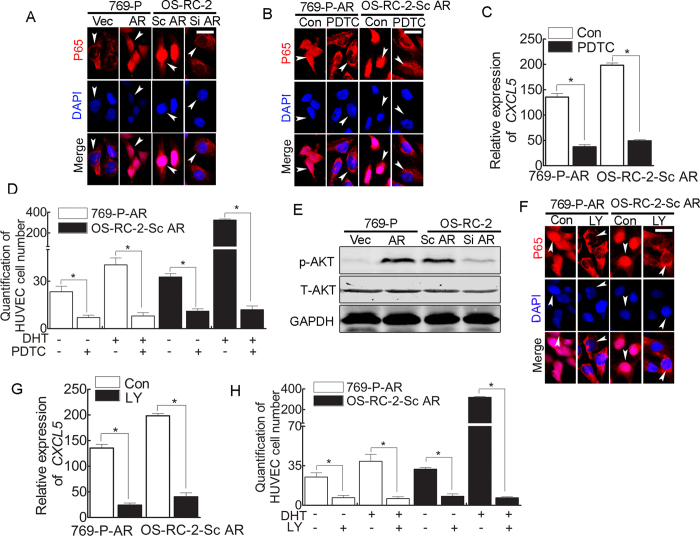
Activated AR signaling in RCC cells leads to activation of the PI3K/AKT/NF-κB pathway. (**A**) Activation of AR in RCC cells led to the nuclear translocation of NF-κB (P65) (immunofluorescence staining). White arrows: positive cells. Bar: 20 μm. (**B**) Immunofluorescent staining indicated that 10 μM PDTC effectively inhibited P65 nuclear translocation. White arrows: positive cells. Bar: 20 μm. (**C**) Q-PCR revealed that NF-κB signaling inhibition with 10 μM PDTC decreased *CXCL5* expression. *P < 0.05. (**D**) Quantification of the Boyden Chamber assay revealed that HUVEC recruitment by AR^+^ RCC cells was attenuated in the presence and/or absence of 10 μM PDTC and 10 nM DHT. *P < 0.05. (**E**) AKT was activated in AR^+^ RCC cells (western blotting). (**F**) Immunofluorescence staining indicated that inhibition of PI3K/AKT signaling by LY (LY294002) in AR^+^ RCC cells resulted in NF-κB pathway inhibition. White arrows: positive cells. Bar: 20 μm. (**G**) Q-PCR revealed that inhibition of PI3K/AKT signaling by LY (LY294002) led to decreased *CXCL5* expression. *P < 0.05; (**H**) Quantification of the Boyden Chamber assay revealed that HUVEC recruitment by AR^+^ RCC cells was attenuated in the presence of LY (LY294002). *P < 0.05.

To study how AR-modulated CXCL5 expression enhances EC recruitment to RCC cells, we focused on NF-κB signaling. NF-κB is a central player controlling inflammation in tumors^22^. First, adding AR to 769-P cells promoted P65 translocation into the nucleus, whereas AR knock-down in OS-RC-2 cells blocked P65 translocation (Fig. 3A). Importantly, interruption approaches using a specific inhibitor of NF-κB (10 μM PDTC) suppressed P65 nuclear translocation (Fig. 3B) and decreased expression of CXCL5 (Fig. 3C) in AR-positive 769-P-AR and OS-RC-2 cells. The consequences of this interruption might lead to decreased recruitment of HUVECs to RCC in the presence or absence of DHT (Fig. 3D). Thus, NF-κB plays a key role in mediating AR-altered CXCL5 expression to enhance EC recruitment to RCC cells.

Next, to dissect the mechanism by which AR modulates NF-κB → CXCL5 signaling, we focused on the PI3K/AKT pathway, as previous studies have demonstrated that PI3K/AKT signaling is aberrantly activated in many cancers, including RCC^23^,^24^,^25^,^26^. Adding AR to 769-P cells increased AKT phosphorylation, and AR knock-down in OS-RC-2 cells decreased AKT activation (Fig. 3E). As expected, a specific inhibitor of AKT (LY294002) inhibited P65 nuclear translocation (Fig. 3F) and decreased CXCL5 expression (Fig. 3G) in AR-positive 769-P-AR and OS-RC-2 cells. Blocking AR-enhanced AKT activation decreased recruitment of HUVECs to RCC in the presence or absence of DHT (Fig. 3H).

The results presented in Fig. 3A–H suggest that AR signaling may function by modulating AKT/NF-κB/CXCL5 signaling to enhance recruitment of ECs to RCC cells.

### Increased ECs in RCC promote RCC cell proliferation, migration and invasion in various RCC cells

**Figure 4:**
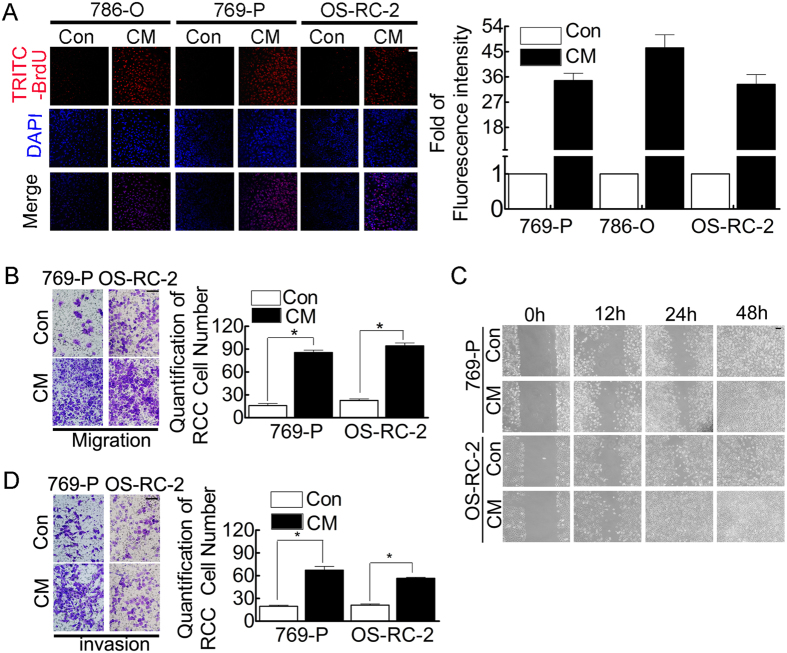
Conditioned medium (CM) from HUVECs co-cultured with RCC cells promotes RCC cell proliferation, migration and invasion. (**A**) RCC proliferation occurred following treatment with CM (BrdU incorporation assay). Left: representative figures. Bar: 100 μm. Right: quantification of the fluorescence intensity. (**B**) RCC migration capacity was enhanced in the presence of CM (Boyden Chamber assay). Left: representative figures. Bar: 100 μm. Right: quantification data. *P < 0.05. (**C**) Wound healing time was decreased in CM-treated RCC cells *vs.* control cells. Bar: 100 μm. (**D**) Boyden Chamber assay: the invasion capacity was enhanced in CM-treated RCC cells. Left: representative figures. Bar: 100 μm. Right: quantification data. *P < 0.05.

To study the consequences of altering the AR-mediated AKT/NF-κB/CXCL5 pathway to enhance EC recruitment to RCC, we first employed BrdU incorporation assays. CM significantly increased cell proliferation in both AR-positive and -negative RCC cells (Fig. 4A).

Using transwell and wound healing migration assays^27^, we also observed that CM significantly increased the migration ability of RCC 769-P and OS-RC-2 cells compared with RCC medium alone (Fig. 4B,C). Similarly, the Matrigel invasion assay^28^ also revealed that CM significantly increased RCC cell invasion abilities (Fig. 4D).

In summary, the results presented in Fig. 4A–D suggest that recruitment of ECs promotes RCC cell proliferation, migration and invasion *in vitro*.

### Increased ECs in RCC promote RCC progression in an *in vivo* mouse model

**Figure 5:**
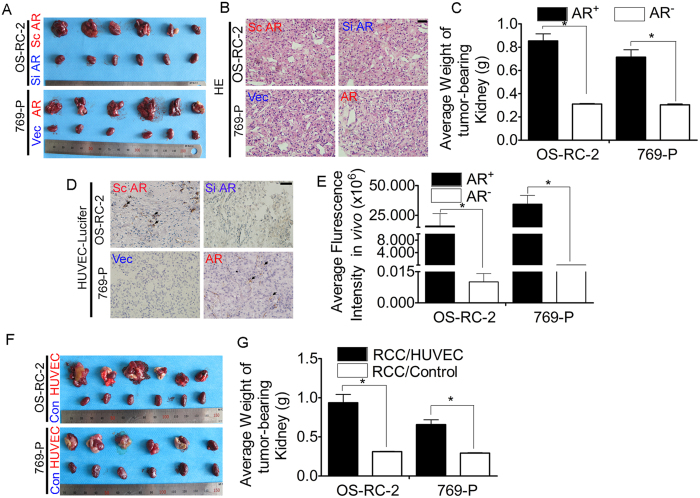
Increased ECs in RCC promotes RCC progression in an *in vivo* mouse model. (**A**) Tumor masses due to orthotropic injection of 769-P-AR/Vec and OS-RC-2 Sc/Si AR cells into mice. (**B**) HE staining: xenografted tissue from tumor-bearing kidneys. Bar: 100 μm. (**C**) Quantification of the average weight of tumor-bearing kidneys resulting from AR^+^ RCC *vs*. AR^−^ RCC grafts. *P < 0.05. (**D**) IHC staining: luciferase-tagged HUVECs within the mouse tumor. Bar: 100 μm. (**E**) Quantification of average luciferase immunofluorescence intensity revealed that the number of luciferase-tagged HUVECs was higher following grafting with AR^+^ RCC cells *versus* AR^−^ RCC cells. *P < 0.05. (**F**) Representative figures of tumor-bearing kidneys by subcapsular injection of RCC cells with or without HUVECs. (**G**) Quantification of the average mass of mouse tumor-bearing kidneys with RCC cells plus HUVECs *versus* RCC cells alone. *P < 0.05.

To confirm the data obtained using *in vitro* cell lines, we first utilized a nude mouse model in which two cell lines (769-P-AR/769-P-Vec cells and OS-RC-2-Si AR/OS-RC-2-Sc AR cells) were orthotopically xenografted into the kidney capsules. Adding AR to the 769-P cells led to increased tumor sizes and masses compared with the control group (Fig. 5A–C). Similar results were also obtained in mice with orthotopically xenografted AR knock-down OS-RC-2 cells; these mice exhibited smaller tumor sizes and masses than the siRNA scramble control mice (Fig. 5A–C), suggesting that AR signaling may promote RCC progression.

To prove that recruitment of ECs to RCC promotes RCC progression, HUVECs tagged with luciferase were injected into the tail vein the day before mice were sacrificed. Adding AR to RCC 769-P cells caused increased HUVEC recruitment than did vector control cells in orthotopically xenografted mice. As expected, AR knock-down in RCC OS-RC-2 cells decreased HUVEC recruitment compared with scramble siRNA cells *in vivo* (Fig. 5D–E).

Importantly, mice with orthotopically xenografted HUVECs plus RCC 769-P or HUVECs plus RCC OS-RC-2 cells also had larger tumor sizes and masses than those with RCC cells alone (Fig. 5F,G), suggesting that ECs promote RCC progression.

Together, the results presented in Fig. 5A–G demonstrate that AR signaling might promote RCC progression in an *in vivo* mouse model through enhanced EC recruitment.

### Nuclear AR expression is positively linked to angiogenesis in RCC patients

**Figure 6:**
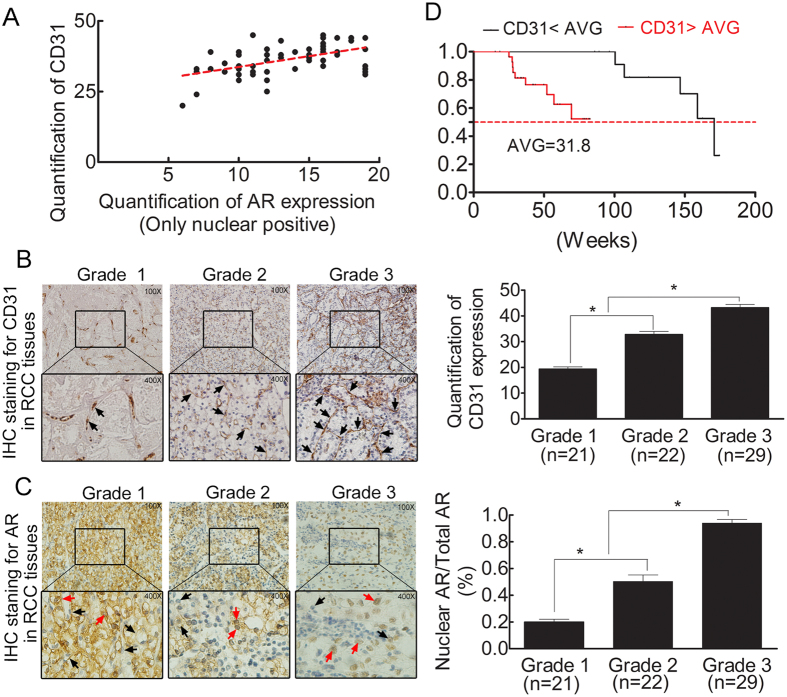
Nuclear AR expression is positively associated with angiogenesis in RCC patients. (**A**) Rank correlation analysis revealed the relationship between CD31 and nuclear AR expression in RCC tissue. (**B**) IHC staining: CD31 (endothelial cell marker, arrows) expression RCC tissues of different grades. Left: representative IHC staining. Upper: 100X; lower: 400X. Right: Quantitative data. *P < 0.05. (**C**) IHC staining for total AR (black arrows) and nuclear AR (red arrows) expression in RCC tissue. Left: representative IHC staining. Upper: 100X; lower: 400X. Right: Quantitative data. *P < 0.05. (**D**) Kaplan-Meier analysis of survival rates for patients with high/low CD31 expression. Patients were divided into two groups according to the mean endothelial cell number per 5 fields (31.8). High CD31 expression is an independent risk factor for patients with RCC.

Finally, to validate the *in vitro* cell line and *in vivo* mouse data, we performed a clinical survey of IHC staining on 72 human RCC samples using antibodies against AR or CD31, the key EC marker during the development of blood vessels that supply nutrition during tumor progression and metastasis^29^. AR nuclear expression was positively correlated with CD31 expression in these 72 human RCC samples (Fig. 6A). Furthermore, the results from this clinical sample survey also revealed CD31 expression to be positively correlated with RCC progression. Higher CD31 expression was observed in higher grade G3 RCC samples than in lower grade G2 or G1 RCC (Fig. 6B). Additionally, the ratio of nuclear AR to total AR was positively related to tumor grade (Fig. 6C).

Altogether, the results presented in Fig. 6A–C confirm the *in vitro* cell line and *in vivo* mouse data. AR expression is positively associated with EC expression, and our clinical survey also indicated that RCC patients with reduced CD31 expression have better survival rates (Fig. 6D). Thus, AR signaling may influence EC expression and possible blood vessel development to promote RCC progression.

## Discussion

RCC is more common among men than women, with a male:female ratio of 1.6:1^30^. In an early study, the incidence of RCC in male mice was found to be 60% *versus* 5% in female mice following carcinogen Fe-NTA treatment, suggesting that gender differences might exist in RCC^31^. Deguchi *et al.*^32^ also observed that androgen treatment increased the incidence of carcinogen Fe-NTA-induced RCC in a rat model. As targeting AR suppresses RCC progression^15^, AR signaling may promote RCC initiation and progression.

Angiogenesis plays a key role in the physiopathology of RCC, and von Hippel-Lindau (VHL) alterations and HIF-2α/VEGF are important mediators of this process^33^. Several strategies have been developed to target angiogenesis for the treatment of mRCC, including inhibition of VEGF receptors (inhibition of tyrosine kinase activity) or VEGF protein binding^33^. Several additional kinase inhibitors, including sunitinib, are used for the treatment of mRCC^34^. However, the relationship between AR signaling and angiogenesis has not been systemically studied. Using *in vitro* and *in vivo* models, we identified a novel mechanism by which AR signaling promotes angiogenesis.

AR expression is detected in almost 30% of RCC cases, and nearly 91% of RCC cells exhibit positive AR staining (mainly in the cytoplasm of renal epithelial cells)^18^. In our study, nuclear AR staining was associated with expression of CD31, suggesting that functional AR is associated with the distribution of EC required for blood vessel development.

PI3K/AKT signaling may play important roles in RCC proliferation and invasion, cancer stem cell maintenance, and angiogenesis within the tumor^23^,^24^,^35^,^36^,^37^. Activation of the PI3K/Akt/mTOR pathway is critical in RCC angiogenesis, and targeting this pathway has been approved for candidate treatment approaches^38^. AR directly influences PI3K/AKT signaling in cancer^39^. In addition, inflammation is also highly related to tumor angiogenesis^40^. NF-κB, the key player in inflammation, may also be involved in recruiting inflammatory immune cells to various tumors^41^ *via* PI3K/AKT signaling^42^. Indeed, we identified PI3K/Akt signaling as a downstream target of AR, which also led to increased CXCL5 expression through P65 translocation. The increased CXCL5 expression in RCC cells enhanced EC recruitment into the tumor microenvironment. CXCL5 may have specific NF-κB binding sites in its promoter region^43^.

Angiogenesis requires endothelial cell migration, a process that is directionally regulated by chemotactic stimuli and further involves degradation of the extracellular matrix to enable progression of the migrating cells^44^. Angiogenesis entails communication between ECs and the surrounding environment, including cancer cells and inflammatory cells^45^. In our study, ECs communicated with RCC cells via CXCL5, which can recruit neutrophils and promote angiogenesis^46^,^47^. In accordance with our study, other researchers have also identified CXCL5 as an important mediator of tumor-derived angiogenesis, and blockade of CXCL5 may be a critical adjunct antiangiogenic therapy against cancer^48^.

In summary, we provide *in vitro* and *in vivo* evidence that AR signaling positively promotes RCC progression by enhancing recruitment of ECs to RCC, which may occur by modulation of AKT → NF-κB → CXCL5 signaling. Targeting this newly identified interaction may facilitate the development of new therapies to slow RCC progression.

## Additional Information

**How to cite this article**: Guan, Z. *et al.* Androgen receptor (AR) signaling promotes RCC progression *via* increased endothelial cell proliferation and recruitment by modulating AKT → NF-κB → CXCL5 signaling. *Sci. Rep.* **6**, 37085; doi: 10.1038/srep37085 (2016).

**Publisher's note**: Springer Nature remains neutral with regard to jurisdictional claims in published maps and institutional affiliations.

## Supplementary Material

Supplementary Information
