# Supplementary material for: Androgen receptor (AR) signaling promotes RCC progression via increased endothelial cell proliferation and recruitment by modulating AKT → NF-κB → CXCL5 signaling
Source: Sci Rep. 2016 Nov 16;6:37085. doi: 10.1038/srep37085 (PMC5111066; doi:10.1038/srep37085)
Supplement: Supplementary Information [file srep37085-s1.pdf]

Androgen receptor (AR) signaling promotes RCC progression *via* increased endothelial cell proliferation and recruitment by modulating AKT→NF-κB→CXCL5 signaling

Zhenfeng Guan[1*], Chong Li[2*],Jinhai Fan[1], Dalin He[1] and Lei Li[1#]

Supplementary Figure S1:

A. Q-PCR-based cytokine array revealed candidate cytokines among 3 sets of RCC cells with transient AR over-expression or knock-down. **B.** Q-PCR revealed *CXCL5* expression in 769-P cells with stable AR expression and in OS-RC-2 cells with stable AR knock-down.

Supplementary Figure S2: Activation of the PI3K/AKT pathway by AR signaling-induced increased expression of CXCL5, which is involved in the NF-KB pathway.

A. Expression of P110 and total P65 (T-P65) was assayed in OSRC-2 and 769-P-AR cells following knock-down of P65 (western blotting). **B.** Expression of P110 and total P65 (T-P65) was assayed in OSRC-2 and 769-P-AR cells following knock-down of P110 (western blotting). **C**. Expression of nuclear P65 (N-P65) was assayed in OSRC-2 and 769-P-AR cells following knock-down of P110 (western blotting). **D**. Real-time PCR revealed expression of CXCL5 in OSRC-2 and 769-P-AR cells following P65 knock-down in the presence of the IGF-1 peptide. *P<0.05. **E**. Real-time PCR revealed expression of CXCL5 in OSRC-2 and 769-P-AR cells after knock-down of P110 in the presence of TNF-α. **P>0.05.

A

Target Genes	786-O PWPI AR/Vec	769-P PWPI AR/Vec	OS-RC-2 PLK ARSC/ARSi
CCL2	12.204	5.6424	0.1092
CCL6	0.9231	1.4762	0.509
CCL11	3.5465	0.6565	0.9981
CXCL1	9.0022	1.0003	0.3234
CXCL2	11.324	10.092	1.5543
CXCL3	4.7308	19.379	1.5323
CXCL4	2.2232	1.8773	0.0343
CXCL5	**16.6**	**69.087**	**35.7633**
CXCL6	12.304	2.3242	6.4064
CXCL7	50.342	2.1339	3.0009
CXCL8	0.0342	0.0034	0.0129
CXCL9	6.7834	0.6561	0.0234
CXCL10	11.485	10.232	0.7734
CXCL11	7.8002	1.0009	1.5008
CXCL12	5.3112	1.5403	1.4002
CXCR1	2.2139	1.3472	11.7609
CXCR2	1.0029	10.658	0.0267
CXCR3	1.9965	0.7898	10.4362
CXCR4	9.2398	1.4587	0.889

B

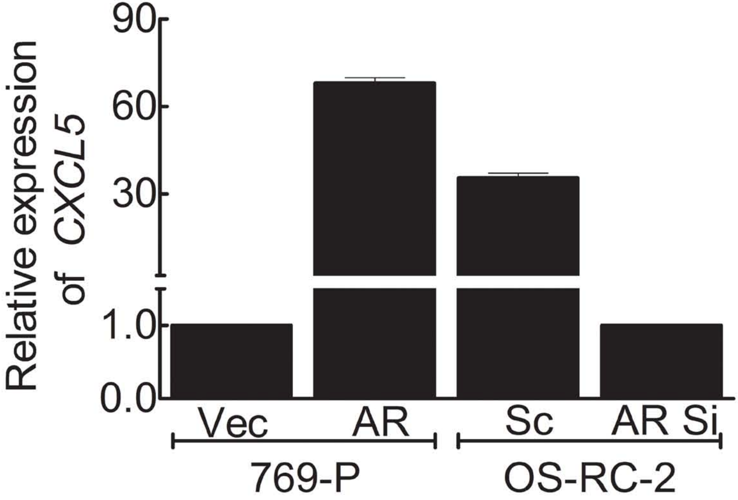

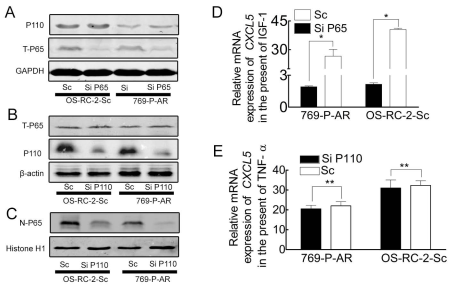
A
P110
T-P65
GAPDH
Sc
Si P65
Si
Si P65
OS-RC-2-Sc
769-P-AR
B
T-P65
P110
β-actin
Sc
Si P110
Sc
Si P110
OS-RC-2-Sc
769-P-AR
C
N-P65
Histone H1
Sc
Si P110
Sc
Si P110
OS-RC-2-Sc
769-P-AR
D
Sc
Si P65
Relative mRNA expression of CXCL5 in the present of IGF-1
45
30
3
0
769-P-AR
OS-RC-2-Sc
E
Si P110
Sc
Relative mRNA expression of CXCL5 in the present of TNF- α
40
30
20
10
0
769-P-AR
OS-RC-2-Sc
